# Unilateral Anomalous Origin of Dorsalis Pedis Artery From Peroneal Artery in a Cadaver

**DOI:** 10.7759/cureus.43414

**Published:** 2023-08-13

**Authors:** Melinda Johnson, Elizabeth A Lavanga, Faisal Aziz

**Affiliations:** 1 Anatomy, Penn State College of Medicine, Hershey, USA; 2 Vascular Surgery, Penn State College of Medicine, Hershey, USA; 3 Cardiac/Thoracic/Vascular Surgery, Penn State Health Milton S. Hershey Medical Center, Hershey, USA

**Keywords:** vascular surgery, cadaver, peroneal artery, anatomic variant, dorsalis pedis artery

## Abstract

In normal anatomy, the anterior tibial artery is typically the first branch of the popliteal artery before it becomes the tibioperoneal trunk. The normal course of the anterior tibial artery includes piercing through the interosseus membrane and continuing through the anterior compartment. It then continues onto the dorsum of the foot as the dorsalis pedis artery at the level of the malleoli. We describe a unique case of an anomalous origin of the dorsalis pedis artery from the peroneal artery. It is important for vascular surgeons to be aware of this variant while interpreting arteriograms of the lower extremity. It can be easily misinterpreted as an occluded distal anterior tibial artery with reconstitution of the dorsalis pedis artery from the collaterals.

## Introduction

The leg, foot, and ankle receive arterial blood from the popliteal artery and its associated branches. A complete understanding of normal anatomy is crucial to the treatment of vascular disease. In the posterior region of the proximal tibiofibular joint, the popliteal artery gives off the anterior tibial artery and continues as the tibioperoneal trunk [1]. The anterior tibial artery pierces the interosseous membrane in the region of the inferior border of the popliteus muscle to enter the anterior compartment of the leg. The anterior tibial artery is situated anterior to the interosseous membrane and is bordered medially by the tibialis anterior muscle and laterally by the extensor hallucis longus muscle.

Distally, the anterior tibial artery passes deep to the extensor retinaculum and continues onto the dorsum of the foot as the dorsalis pedis artery (DPA) at the level of the malleoli. This location also serves as an anatomical landmark for pulse palpation [2]. The DPA then gives rise to the medial and lateral tarsal arteries as well as the arcuate artery distally. The arcuate artery passes laterally over the base of the metatarsals and anastomoses with the lateral tarsal artery. The DPA continues distally towards the first intermetatarsal space. Prior to passing deep between the heads of the first dorsal interosseous muscle, the first dorsal metatarsal artery branches from DPA. The DPA and first dorsal metatarsal artery give rise to cutaneous branches, which supply the medial dorsal foot. Finally, the DPA completes the plantar arterial arch and provides the first plantar metatarsal artery [3].

In addition to a robust understanding of normal lower extremity vascular anatomy, an awareness of the anatomic variants such as those described in this report, is necessary for application in vascular, orthopedic, and radiologic medicine. In this report, we describe an anomalous origin of the dorsalis pedis artery from the peroneal artery which holds important clinical implications including interpretation of arteriograms, estimation of disease burden in peripheral arterial disease, and revascularization planning.

## Case presentation

**Figure 1:**
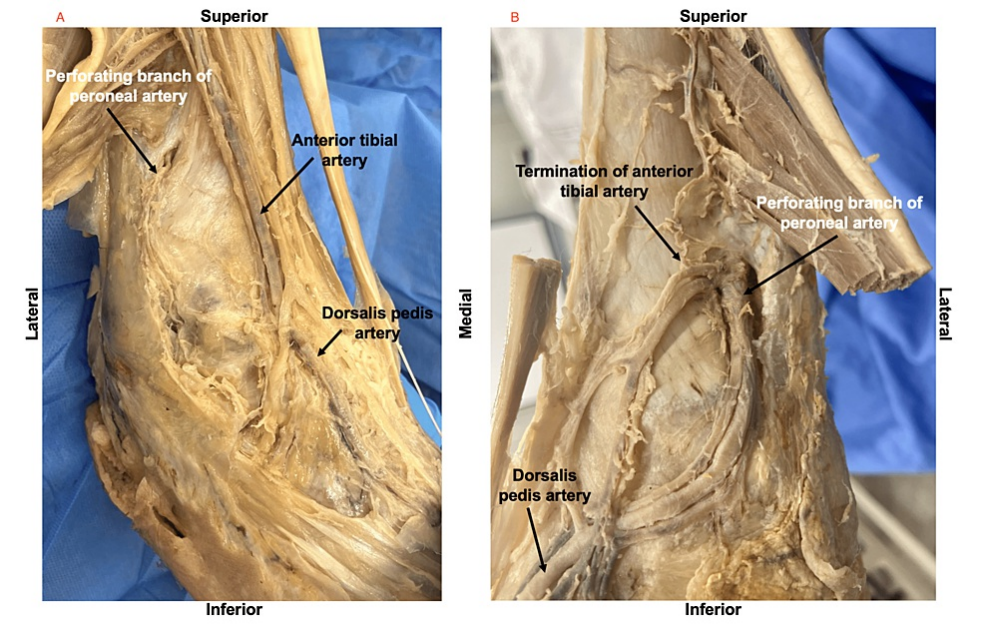
Anterolateral View of Normal Right Leg (A) and Variation Left Leg (B) Showing Termination of the Anterior Tibial Artery and Continuation of the Peroneal Artery Perforating Branch as the DPA DPA: Dorsalis Pedis Artery

Cadaveric dissection of a 94-year-old male who died of end-stage cerebral infarction revealed a unilateral anomalous origin of the right dorsalis pedis artery. Examination of the lower extremity revealed no scars suggestive of prior operations. The right popliteal artery entered the posterior compartment of the leg from the popliteal fossa and gave rise to the right anterior tibial artery and the right tibioperoneal trunk. The path of the right anterior tibial artery was traced through the proximal oval aperture in the interosseous membrane to reach the anterior compartment of the leg (Figure 1). 

**Figure 2:**
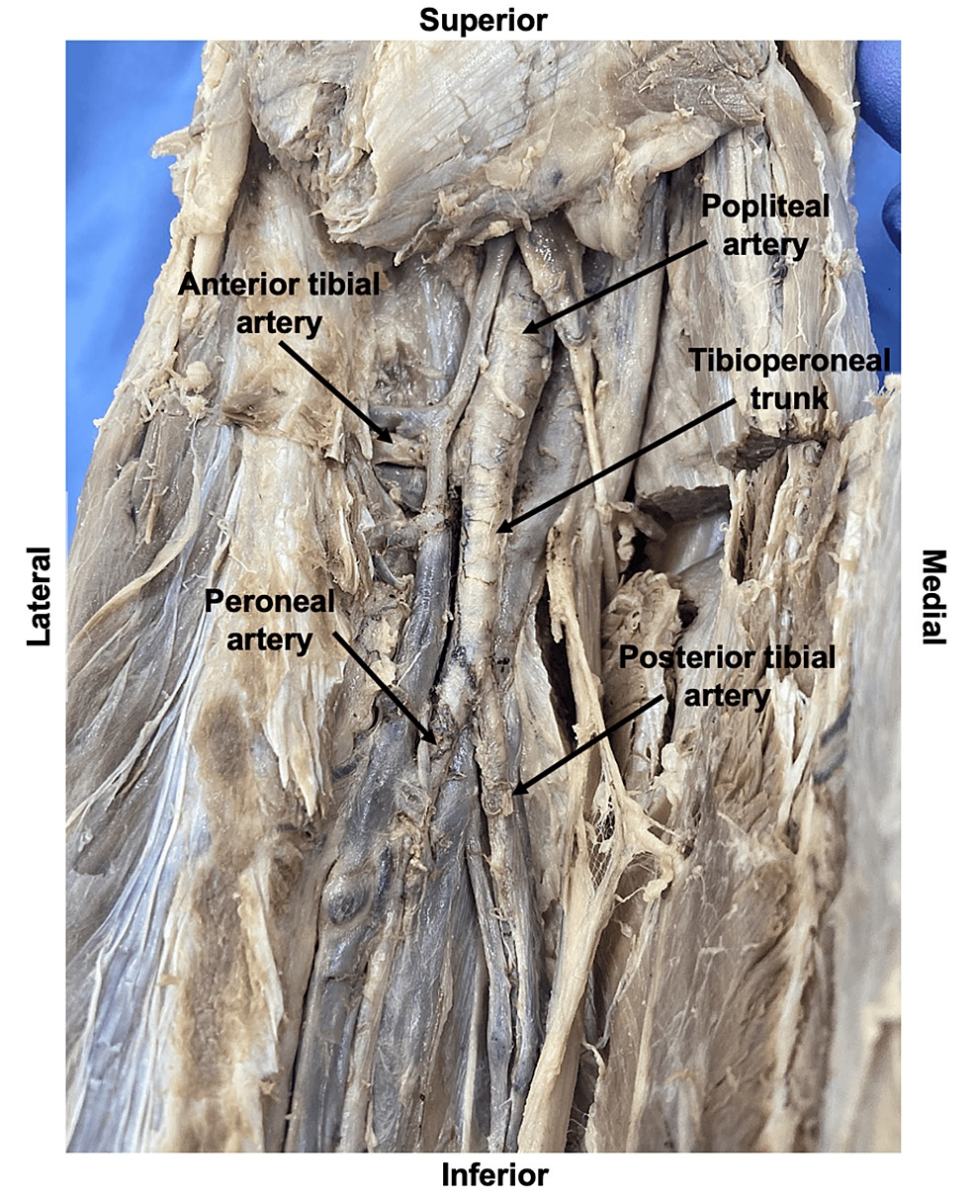
Posterior View of Branching of the Anterior Tibial Artery and Tibioperoneal Trunk from the Popliteal Artery

The right tibioperoneal trunk provided the right posterior tibial artery medially and the right peroneal artery laterally. The right anterior tibial artery entered the anterior compartment of the leg and was identified intermediate to tibialis anterior muscle and extensor hallucis longus muscle running longitudinally with the right anterior tibial veins and the right deep peroneal nerve. The right anterior tibial artery became hypoplastic and terminated as muscular and fascial branches. The right peroneal artery descended in the posterior compartment of the leg deep to the transverse intermuscular septum. It was then visualized passing through the distal oval aperture in the interosseous membrane to enter the anterior compartment of the leg as the perforating branch of the peroneal artery. The right perforating branch of the peroneal artery bifurcated into a smaller medial branch and a larger lateral branch. The medial branch was seen anastomosing with the anterior tibial artery. This is where the anterior tibial artery becomes hypoplastic and terminates. The lateral branch passed deep to the tendons of fibularis tertius and extensor digitorum longus muscles to continue as the right DPA (Figure 2). 

The right DPA continued distally alongside the right dorsalis pedis veins and the right deep peroneal nerve on the dorsum of the right foot.

## Discussion

Anatomical variations of the DPA

Variations have been identified in the origin, course, and branching pattern of the DPA. Anomalous origin of DPA from the perforating branch of the peroneal artery has been described before in both cadaveric and arteriogram studies. According to Hemamalini and Manjunatha, an anomalous origin of DPA can occur bilaterally or unilaterally [4]. Three identified variations include lateral deviation, bifurcation or trifurcation of DPA, and absence of the arcuate artery. In the case of a bilateral or unilateral anomalous origin of DPA, an enlarged perforating branch of the peroneal artery continues as DPA after piercing the interosseous membrane. Deviation and bifurcation of DPA have been described as the anterior tibial artery continuing as DPA and the DPA quickly dividing into a medial and lateral branch on the lateral dorsum of the foot. It is typical for the medial branch of DPA to provide the tarsal arteries, arcuate artery, first dorsal metatarsal artery, and deep plantar artery. The lateral branch becomes hypoplastic and cannot be traced. Hemamalini and Manjunatha noted that the arcuate artery was absent in seven out of 27 limbs. This resulted in the branching of the second, third, and fourth dorsal metatarsal arteries from the DPA [4].

Vazquez et al. studied the blood supply of the foot and ankle using a sample of 150 human embalmed cadavers. It was noted that 287 cases (95.7%) displayed a normal continuation of the anterior tibial artery to DPA distal to the talocrural joint. In these cases, the DPA was identified lateral to the tendon of the extensor hallucis longus and medial to the tendons of the extensor digitorum longus. Six cases (2%) demonstrated a lateral deviation of the anterior tibial artery as far as the lateral malleolus. They reported an enlarged perforating branch of the peroneal artery continuing as DPA in four out of 300 limbs, for an incidence rate of 1.3%. Lastly, three cases (1%) demonstrated the origination of an additional lateral branch from the anterior tibial artery that replaced the perforating branch of the peroneal artery [5].

This anomaly was additionally studied by Keen via cadaveric dissection of 140 subjects, or 280 limbs, and anomalous origin was discovered unilaterally 12 times, and bilaterally once, demonstrating 5% incidence [6]. Kim et al. performed femoral arteriograms on 1000 extremities. Of these, 495 extremities were analyzed for arterial supply to the foot. Eight of 495 subjects (1.6%) were identified to have the perforating branch of the peroneal artery continue as DPA with the anterior tibial artery becoming hypoplastic proximal to the ankle [7]. In a study by Vijayalakshmi et al., 50 lower limbs were dissected and four were found to have DPA arise as a continuation of the perforating branch of the peroneal artery (8%) [8]. Finally, the Huber study dissected 200 feet and found six (3%) that presented with this anatomical variation. An anomalous origin of DPA from the perforating branch of the peroneal artery in the presence of a hypoplastic anterior tibial artery occurs in approximately 1.3-8% of limbs [5-9].

Clinical implications

Awareness of anatomical variations in the origin, course, and branching patterns of DPA is important for vascular surgeons, orthopedic surgeons, and radiologists. Documentation of dorsalis pedis pulse via palpation or Doppler is useful in evaluating anterior compartment syndrome and peripheral arterial diseases (PAD) [4,10]. However, a diminished pulse is not always indicative of the aforementioned pathologies and may instead be due to an anomaly of the DPA. Furthermore, an understanding of the lower extremity arterial anatomy of individual patients is necessary for the successful completion and assessment of the patency of proximal and distal bypass grafts as well as other revascularization procedures. It is also important to keep this anomaly in mind in the interpretation of leg arteriograms, as it may falsely lead to the impression that the distal anterior tibial artery is occluded and DPA is reconstituting from collaterals. Lastly, the DPA and its branches contribute to the dorsalis pedis fasciocutaneous flap, which is used for soft tissue reconstruction in the lower leg, dorsum of the hand, oral, and nasal cavities [11-13]. Imaging modalities such as angiograms may be utilized to obtain a more complete picture of anatomical variation in patients with peripheral arterial disease and will supplement the checking of pedal pulses, avoiding overestimation of disease while providing a schematic for revascularization planning when deemed necessary.

Embryonic origin

Variations in the branching pattern of foot and ankle vasculature can be attributed to disruption of normal embryonic development [14]. The developing lower limb bud receives blood from the primary (sciatic) artery and the femoral artery. These two vessels join in the popliteal fossa to form the popliteal artery. In adults, the sciatic artery regresses and certain sections persist as the popliteal artery, peroneal artery, and accompanying artery of the sciatic nerve [15]. The anterior and posterior tibial arteries are derived from the femoral artery. Thus, the anterior tibial and peroneal arteries arise from different vessels during development. Therefore, regression and persistence of the sciatic artery and its connections with the femoral artery could explain the unilateral anomalous origin of the DPA from the perforating branch of the peroneal artery and hypoplasia of the anterior tibial artery seen here [4].

## Conclusions

Even though anomalous variations in lower extremity vascular anatomy have been described in the literature, they have been largely ignored within medical education. Vascular practitioners are often faced with recognition and treatment of anomalous vascular anatomy without adequate background education or understanding of the possible variants. This report describes a key variation of lower extremity vasculature by highlighting the peroneal origin of the DPA. Recognition of this variant has numerous clinical applications including radiologic interpretation of arteriogram studies, palpation, and bedside Doppler of pulses, and planning of revascularization efforts.
